# *N*′-(4-Fluoro­benzyl­idene)acetohydrazide

**DOI:** 10.1107/S1600536810042765

**Published:** 2010-10-30

**Authors:** Huan-mei Guo, Li Liu, Jie Yang, Yang-chun Liu

**Affiliations:** a Microscale Science Institute, Weifang University, Weifang 261061, People’s Republic of China; b Department of Chemistry and Chemical Engineering, Weifang University, Weifang 261061, People’s Republic of China

## Abstract

The title compound, C_9_H_9_FN_2_O, was prepared by the reaction of 4-fluoro­benzophenone and acethydrazide. In the mol­ecule, all non-H atoms are essentially coplanar [r.m.s. deviation = 0.065 (2) Å]. In the crystal, mol­ecules are linked into centrosymmetric dimers by pairs of inter­molecular N—H⋯O hydrogen bonds.

## Related literature

For general background to Schiff bases, see: Goswami *et al.* (2009 ▶); Zhang *et al.* (2010 ▶). For related structures, see: Li & Jian (2008 ▶); Girgis (2006 ▶); Yang *et al.* (2010 ▶);
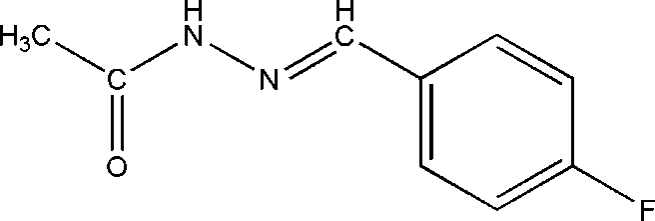


## Experimental

### 

#### Crystal data

C_9_H_9_FN_2_O
*M* *_r_* = 180.18
Monoclinic, 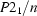

*a* = 10.443 (2) Å
*b* = 4.0418 (8) Å
*c* = 21.172 (4) Å
β = 96.71 (3)°
*V* = 887.5 (3) Å^3^
*Z* = 4
Mo *K*α radiation
μ = 0.11 mm^−1^
*T* = 293 K
0.24 × 0.22 × 0.22 mm

#### Data collection

Bruker SMART CCD diffractometer
7536 measured reflections
2033 independent reflections
1412 reflections with *I* > σ(*I*)
*R*_int_ = 0.025

#### Refinement

*R*[*F* ^2^ > 2σ(*F* ^2^)] = 0.052
*wR*(*F* ^2^) = 0.176
*S* = 1.12
2033 reflections
118 parameters
H-atom parameters constrained
Δρ_max_ = 0.31 e Å^−3^
Δρ_min_ = −0.23 e Å^−3^

### 

Data collection: *SMART* (Bruker, 1997 ▶); cell refinement: *SAINT* (Bruker, 1997 ▶); data reduction: *SAINT*; program(s) used to solve structure: *SHELXS97* (Sheldrick, 2008 ▶); program(s) used to refine structure: *SHELXL97* (Sheldrick, 2008 ▶); molecular graphics: *SHELXTL* (Sheldrick, 2008 ▶); software used to prepare material for publication: *SHELXTL*.

## Supplementary Material

Crystal structure: contains datablocks global, I. DOI: 10.1107/S1600536810042765/lh5151sup1.cif

Structure factors: contains datablocks I. DOI: 10.1107/S1600536810042765/lh5151Isup2.hkl

Additional supplementary materials:  crystallographic information; 3D view; checkCIF report

## Figures and Tables

**Table 1:** Hydrogen-bond geometry (Å, °)

*D*—H⋯*A*	*D*—H	H⋯*A*	*D*⋯*A*	*D*—H⋯*A*
N1—H1*A*⋯O1^i^	0.86	2.04	2.899 (2)	176

Symmetry code: (i) 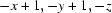.

**Fig. 1.**
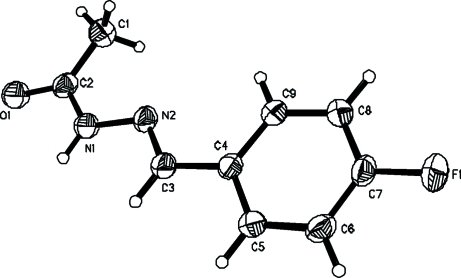
The molecular structure of the title compound with the atom-labeling scheme. Displacement ellipsoids are drawn at the 30% probability level.

## supplementary crystallographic information

### Comment

Schiff bases have received considerable attention in the literature (Zhang *et al.*, 2010; Goswami *et al.*, 2009). As part of our search for new schiff base compounds we synthesized the title compound(I) and its crystal structure is reported herein. In the title compound (Fig. 1), the bond lengths and angles are similar to those in related structures (Li & Jian, 2008; Yang *et al.*, 2010). The C3═N2 bond length of 1.269 (2)Å is slight shorter than the C═N double bond [1.281 (2) Å and 1.2732 (18)] reported by Girgis (2006) and Yang *et al.* (2010). In the crystal structure, molecules are linked into centrosymmetric dimers by pairs of intermolecular N—H···O hydrogen bonds (Table 1).

### Experimental

A mixture of the 4-fluorobenzophenone (0.02 mol) and acethydrazide (0.02 mol) was stirred in refluxing ethanol (30 ml) for 2 h to afford the title compound (yield 65%). Single crystals suitable for X-ray measurements were obtained by recrystallization from a solution of the title compound in ethanol at room temperature.

### Refinement

All H atoms were fixed geometrically and allowed to ride on their attached atoms, with C—H 0.93–0.96Å, N—H = 0.86 Å and *U*_iso_(H) = 1.2*U*_eq_(C,N) or 1.5*U*_eq_(C_methyl_)

### Crystal data

**Table:**

C_9_H_9_FN_2_O	*F*(000) = 376
*M**_r_* = 180.18	*D*_x_ = 1.349 Mg m^−^^3^
Monoclinic, *P*2_1_/*n*	Mo *K*α radiation, λ = 0.71073 Å
Hall symbol: -P 2yn	Cell parameters from 2033 reflections
*a* = 10.443 (2) Å	θ = 3.7–27.5°
*b* = 4.0418 (8) Å	µ = 0.11 mm^−^^1^
*c* = 21.172 (4) Å	*T* = 293 K
β = 96.71 (3)°	Bar, colourless
*V* = 887.5 (3) Å^3^	0.24 × 0.22 × 0.22 mm
*Z* = 4	

### Data collection

**Table:**

Bruker SMART CCD diffractometer	1412 reflections with *I* > σ(*I*)
Radiation source: fine-focus sealed tube	*R*_int_ = 0.025
graphite	θ_max_ = 27.5°, θ_min_ = 3.3°
φ and ω scans	*h* = −13→13
7536 measured reflections	*k* = −5→5
2033 independent reflections	*l* = −27→27

### Refinement

**Table:**

Refinement on *F*^2^	Primary atom site location: structure-invariant direct methods
Least-squares matrix: full	Secondary atom site location: difference Fourier map
*R*[*F*^2^ > 2σ(*F*^2^)] = 0.052	Hydrogen site location: inferred from neighbouring sites
*wR*(*F*^2^) = 0.176	H-atom parameters constrained
*S* = 1.12	*w* = 1/[σ^2^(*F*_o_^2^) + (0.1001*P*)^2^ + 0.0565*P*] where *P* = (*F*_o_^2^ + 2*F*_c_^2^)/3
2033 reflections	(Δ/σ)_max_ < 0.001
118 parameters	Δρ_max_ = 0.31 e Å^−^^3^
0 restraints	Δρ_min_ = −0.23 e Å^−^^3^

### Special details

**Table:**

Geometry. All e.s.d.'s (except the e.s.d. in the dihedral angle between two l.s. planes) are estimated using the full covariance matrix. The cell e.s.d.'s are taken into account individually in the estimation of e.s.d.'s in distances, angles and torsion angles; correlations between e.s.d.'s in cell parameters are only used when they are defined by crystal symmetry. An approximate (isotropic) treatment of cell e.s.d.'s is used for estimating e.s.d.'s involving l.s. planes.
Refinement. Refinement of *F*^2^ against ALL reflections. The weighted *R*-factor *wR* and goodness of fit *S* are based on *F*^2^, conventional *R*-factors *R* are based on *F*, with *F* set to zero for negative *F*^2^. The threshold expression of *F*^2^ > σ(*F*^2^) is used only for calculating *R*-factors(gt) *etc*. and is not relevant to the choice of reflections for refinement. *R*-factors based on *F*^2^ are statistically about twice as large as those based on *F*, and *R*- factors based on ALL data will be even larger.

### Fractional atomic coordinates and isotropic or equivalent isotropic displacement parameters (Å^2^)

**Table:**

	*x*	*y*	*z*	*U*_iso_*/*U*_eq_	
N2	0.41309 (13)	0.8047 (4)	0.12439 (6)	0.0506 (4)	
C4	0.50404 (14)	0.7947 (4)	0.23307 (7)	0.0448 (4)	
C3	0.50479 (15)	0.7172 (4)	0.16566 (7)	0.0517 (4)	
H3A	0.5741	0.6012	0.1527	0.062*	
N1	0.42670 (14)	0.7124 (4)	0.06305 (6)	0.0574 (4)	
H1A	0.4921	0.5941	0.0561	0.069*	
O1	0.35986 (12)	0.7130 (4)	−0.04043 (5)	0.0692 (4)	
F1	0.50984 (11)	0.9900 (3)	0.42320 (4)	0.0807 (4)	
C2	0.34069 (16)	0.8017 (4)	0.01332 (7)	0.0533 (4)	
C8	0.40253 (16)	1.0213 (4)	0.31985 (7)	0.0545 (4)	
H8A	0.3333	1.1268	0.3352	0.065*	
C7	0.50862 (17)	0.9235 (4)	0.36057 (7)	0.0536 (4)	
C5	0.60833 (15)	0.6986 (4)	0.27603 (7)	0.0518 (4)	
H5A	0.6772	0.5882	0.2613	0.062*	
C9	0.40169 (14)	0.9588 (4)	0.25596 (7)	0.0497 (4)	
H9A	0.3318	1.0270	0.2277	0.060*	
C6	0.61129 (16)	0.7648 (4)	0.34044 (7)	0.0557 (4)	
H6A	0.6815	0.7025	0.3691	0.067*	
C1	0.22542 (17)	0.9959 (4)	0.02534 (8)	0.0631 (5)	
H1B	0.1738	1.0404	−0.0143	0.095*	
H1C	0.2522	1.2011	0.0456	0.095*	
H1D	0.1757	0.8720	0.0525	0.095*	

### Atomic displacement parameters (Å^2^)

**Table:**

	*U*^11^	*U*^22^	*U*^33^	*U*^12^	*U*^13^	*U*^23^
N2	0.0517 (8)	0.0632 (8)	0.0366 (7)	−0.0062 (6)	0.0045 (5)	−0.0048 (5)
C4	0.0445 (8)	0.0502 (8)	0.0393 (8)	−0.0073 (6)	0.0030 (6)	−0.0030 (6)
C3	0.0471 (8)	0.0647 (10)	0.0433 (8)	−0.0038 (7)	0.0058 (6)	−0.0067 (7)
N1	0.0497 (8)	0.0851 (10)	0.0374 (7)	0.0001 (7)	0.0045 (5)	−0.0076 (6)
O1	0.0619 (7)	0.1067 (11)	0.0384 (6)	−0.0006 (7)	0.0029 (5)	−0.0048 (6)
F1	0.0863 (8)	0.1149 (10)	0.0392 (6)	0.0063 (7)	0.0000 (5)	−0.0137 (5)
C2	0.0511 (9)	0.0682 (10)	0.0400 (8)	−0.0107 (8)	0.0033 (6)	−0.0005 (7)
C8	0.0513 (9)	0.0650 (10)	0.0472 (9)	0.0041 (7)	0.0059 (6)	−0.0074 (7)
C7	0.0600 (9)	0.0648 (10)	0.0350 (7)	−0.0067 (8)	0.0017 (6)	−0.0056 (7)
C5	0.0432 (8)	0.0635 (10)	0.0484 (8)	0.0001 (7)	0.0035 (6)	−0.0029 (7)
C9	0.0451 (8)	0.0584 (9)	0.0441 (8)	−0.0002 (7)	−0.0011 (6)	−0.0030 (6)
C6	0.0488 (9)	0.0697 (10)	0.0458 (8)	−0.0026 (7)	−0.0061 (6)	0.0018 (7)
C1	0.0681 (11)	0.0673 (11)	0.0533 (9)	0.0041 (9)	0.0043 (7)	0.0044 (8)

### Geometric parameters (Å, °)

**Table:**

N2—C3	1.269 (2)	C8—C9	1.375 (2)
N2—N1	1.3744 (17)	C8—C7	1.380 (2)
C4—C5	1.390 (2)	C8—H8A	0.9300
C4—C9	1.392 (2)	C7—C6	1.360 (2)
C4—C3	1.462 (2)	C5—C6	1.386 (2)
C3—H3A	0.9300	C5—H5A	0.9300
N1—C2	1.351 (2)	C9—H9A	0.9300
N1—H1A	0.8600	C6—H6A	0.9300
O1—C2	1.2317 (17)	C1—H1B	0.9600
F1—C7	1.3516 (17)	C1—H1C	0.9600
C2—C1	1.484 (2)	C1—H1D	0.9600
			
C3—N2—N1	114.97 (14)	F1—C7—C8	118.09 (15)
C5—C4—C9	118.69 (14)	C6—C7—C8	122.96 (14)
C5—C4—C3	119.05 (15)	C6—C5—C4	121.01 (15)
C9—C4—C3	122.25 (14)	C6—C5—H5A	119.5
N2—C3—C4	121.53 (15)	C4—C5—H5A	119.5
N2—C3—H3A	119.2	C8—C9—C4	120.87 (14)
C4—C3—H3A	119.2	C8—C9—H9A	119.6
C2—N1—N2	122.09 (15)	C4—C9—H9A	119.6
C2—N1—H1A	119.0	C7—C6—C5	118.13 (15)
N2—N1—H1A	119.0	C7—C6—H6A	120.9
O1—C2—N1	118.51 (16)	C5—C6—H6A	120.9
O1—C2—C1	122.36 (15)	C2—C1—H1B	109.5
N1—C2—C1	119.12 (14)	C2—C1—H1C	109.5
C9—C8—C7	118.32 (15)	H1B—C1—H1C	109.5
C9—C8—H8A	120.8	C2—C1—H1D	109.5
C7—C8—H8A	120.8	H1B—C1—H1D	109.5
F1—C7—C6	118.94 (15)	H1C—C1—H1D	109.5

### Hydrogen-bond geometry (Å, °)

**Table:**

*D*—H···*A*	*D*—H	H···*A*	*D*···*A*	*D*—H···*A*
N1—H1A···O1^i^	0.86	2.04	2.899 (2)	176

Symmetry codes: (i) −*x*+1, −*y*+1, −*z*.
